# Biomedical relation extraction via knowledge-enhanced reading comprehension

**DOI:** 10.1186/s12859-021-04534-5

**Published:** 2022-01-06

**Authors:** Jing Chen, Baotian Hu, Weihua Peng, Qingcai Chen, Buzhou Tang

**Affiliations:** 1 Intelligent Computing Research Center, Harbin Institute of Technology (Shenzhen), Shenzhen, China; 2 Baidu International Technology (Shenzhen) Co., Ltd, Shenzhen, China; 3 Peng Cheng Laboratory, Shenzhen, China

**Keywords:** Biomedical relation extraction, Reading comprehension, Knowledge attention mechanism

## Abstract

**Background:**

In biomedical research, chemical and disease relation extraction from unstructured biomedical literature is an essential task. Effective context understanding and knowledge integration are two main research problems in this task. Most work of relation extraction focuses on classification for entity mention pairs. Inspired by the effectiveness of machine reading comprehension (RC) in the respect of context understanding, solving biomedical relation extraction with the RC framework at both intra-sentential and inter-sentential levels is a new topic worthy to be explored. Except for the unstructured biomedical text, many structured knowledge bases (KBs) provide valuable guidance for biomedical relation extraction. Utilizing knowledge in the RC framework is also worthy to be investigated. We propose a knowledge-enhanced reading comprehension (KRC) framework to leverage reading comprehension and prior knowledge for biomedical relation extraction. First, we generate questions for each relation, which reformulates the relation extraction task to a question answering task. Second, based on the RC framework, we integrate knowledge representation through an efficient knowledge-enhanced attention interaction mechanism to guide the biomedical relation extraction.

**Results:**

The proposed model was evaluated on the BioCreative V CDR dataset and CHR dataset. Experiments show that our model achieved a competitive document-level F1 of 71.18% and 93.3%, respectively, compared with other methods.

**Conclusion:**

Result analysis reveals that open-domain reading comprehension data and knowledge representation can help improve biomedical relation extraction in our proposed KRC framework. Our work can encourage more research on bridging reading comprehension and biomedical relation extraction and promote the biomedical relation extraction.

## Background

**Fig. 1:**
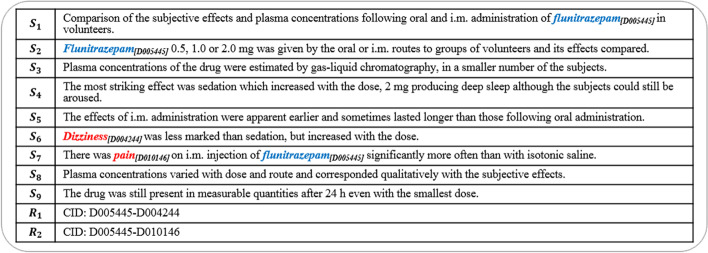
The sample document. Chemical and disease mentions are marked in blue and red, respectively. CID means the chemical-induced disease relation

Chemical, disease, and their relations play an important role in biomedical research [1] and relation extraction is an essential task in biomedical text information extraction. Many experts have been making efforts to perform research on automatic biomedical information extraction from unstructured text. To promote research on chemical-disease relation (CDR) extraction, the BioCreative-V community proposed a subtask: chemical-induced disease (CID) relation extraction. Additionally, [2] proposed a document-level dataset for chemical reaction (CHR) relation extraction. Here, the relations between entities are expressed not only in a single sentence but also across sentences. As described by [3], 30% of relations in the Biocreative V CDR data are expressed across more than one sentence. As an example in Fig. 1, it shows the title and abstract of a document containing two chemical-induced disease pairs (D005445, D004244) and (D005445, D010146). Among these instances, chemical *‘flunitrazepam’* and disease *‘pain’* appear in the same sentence, while chemical *‘flunitrazepam’* and disease *‘dizziness’* are expressed across sentence boundaries.

Typically, relation extraction can be formulated as a classification task for candidate entity pairs, and many machine learning methods have been investigated to score mention pairs to extract relations, including traditional machine learning (ML) methods and neural network (NN)-based methods. Most of them attempt to mine the context information between entity mention pairs to provide evidence for relation extraction. Some extract rich statistical and knowledge features, some mark the entities by start and end symbols [4, 5] or extract the shortest dependency path between entities [1, 6, 7]. It helps to capture the context information and make up the ability to model long-distance context sequences. Early studies mainly utilized maximum entropy (ME) models, support vector machines(SVMs)and other kernel-based models combined with rich context features (e.g., statistical linguistic features), knowledge features and graph structures [8–10]. Li et al. [10] also exploits co-training with additional unlabeled training data. Since feature extraction is time-consuming and difficult to expand, neural network-based methods are widely explored and achieve significant performance. Le et al. [6] extracts the shortest dependency path (SDP) and learned context information through Convolutional Neural Network (CNN) for CID extraction. Nguyen et al. [11] investigates the incorporation of character-based word representations into a standard CNN-based relation extraction model. Verga et al. [3] forms pairwise predictions over entire abstracts using a self-attention encoder. Zheng et al. [4] uses CNN and LSTM to learn the document semantic formation and integrated knowledge representation. Li et al. [5] utilizes recurrent piecewise convolutional neural networks integrating knowledge features. Sahu et al. [2] proposes to build a labeled edge graph convolutional neural network on a document to capture local and non-local context dependency information for inter-sentence biomedical relation extraction. Zhou et al. [1] proposes a knowledge-guided convolution network to leverage prior knowledge representation on the SDP sequence for CID extraction.

Machine reading comprehension (MRC) aims to answer a query according to its corresponding contexts, one of which is to extract answer spans from contexts. The task is formulated as a multi-classification task to classify the start index and the end index of the answer over its contexts. Inspired by the performance and the comprehension ability, MRC has been a trend to solve other natural language processing (NLP) tasks. Levy et al. [12] reduces the zero-shot relation to the problem of answering simple reading comprehension questions to potentially extract facts of new types that were neither specified nor observed a priori. Li et al. [13] casts the entity-relation task as a multi-turn question answering problem and identifies the answer spans from the context. Li et al. [14] proposes to formulate the flat and nested named entity recognition problems as a machine reading comprehension task instead of a sequence labeling task. Additionally, tasks such as summarization, machine translation and so on are framed as question answering by making task specifications to take the form of a question, a context and an answer [15].

Motivated by the capability of context understanding on documents, we regard biomedical relation extraction as a reading comprehension problem. We utilize a question formulated by the chemical and relation description to query the context for diseases or chemicals, hence acquiring the relation between chemical and disease entities or the relation between chemical entities. In this paper, we are interested in handling biomedical relation extraction with the reading comprehension framework based on the efficient pretrained language model (LM), effectively integrating knowledge with context together and distinguishing different knowledge in this framework. Hence, we propose a knowledge-enhanced RC (KRC) framework for biomedical relation extraction, which integrates knowledge by effective two-step attention layers. The proposed method was evaluated on the BioCreative V CDR dataset and the CHR dataset respectively. Experiments show that our proposed model achieved competitive performance on both datasets compared with other state-of-the-art methods. Our contributions are as follows:

- To the best of our knowledge, this paper first proposes a novel reading comprehension (RC) framework to address the biomedical relation extraction from the literature. Our work may encourage more research on bridging MRC and biomedical relation extraction so as to take advantage of MRC.
- To make full use of the pretrained language model (LM) and knowledge representation, this paper proposes a knowledge-enhanced RC model based on pretrained LMs to improve biomedical relation extraction.
- Through experiments, we demonstrate the effectiveness of using open-domain reading comprehension data and knowledge information in our proposed RC framework for biomedical relation extraction. We show that our method can achieve competitive performance on two document-level datasets.

## Problem formulation

Given a context sequence $C=(w_c^1, w_c^2, \ldots , w_c^n)$ and two entities $e_1=(w_{c}^{s_{e1}}, w_{c}^{s_{e1}+1}, \ldots , w_{c}^{e_{e1}})$ and $e_2=(w_{c}^{s_{e2}}, w_{c}^{s_{e2}+1}, \ldots , w_{c}^{e_{e2}})$ in the context, relation extraction(RE) aims to clarify the relation *r* between $e_1$ and $e_2$, where $r \in R$ is selected from a predefined relation list *R*. For the chemical-induced disease (CID) relation extraction task, the relation *r* is ‘*induce*’. Here, we reduce the relation extraction task as a reading comprehension task with unanswerable answers. We transform the annotated RE data $(Context, Entity\ e_1, Entity\ e_2, relation\ r)$ to the RC data $(Context, Query(e_1,r), Answer\ e_2)$. Given the context sequence $C=(w_c^1, w_c^2, \ldots , w_c^n)$, the entity $e_1=(w_{c}^{s_{e1}}, w_{c}^{s_{e1}+1}, \ldots , w_{c}^{e_{e1}})$ and the relation *r*, extract the entity $e_2=(w_{c}^{s_{e2}}, w_{c}^{s_{e2}+1}, \ldots , w_{c}^{e_{e2}})$ from the context by answering a query $Q=(w_q^1, w_q^2, \ldots , w_q^m)$ constructed by $e_1$ and relation *r* description. $s_{e2}$ and $e_{e2}$ respectively denote the start index and the end index, where $s_{e2} \in [1,n]$, $e_{e2} \in [1,n]$ and $s_{e2} \le e_{e2}$.

Given context *C* and question *Q*, our method either returns an answer span or indicates that there is no answer.

## Methods

We adopt a competitive pretrained language model BERT [16] as our backbone that deals with SQuAD [17] and suits the condition of no answer. Our model consists of three major layers: (1) BERT encoder layer; (2) knowledge-enhanced attention layer; (3) prediction layer. Details are described as follows.

### BERT encoder layer

To be in line with BERT, given the context sequence $C=(w_c^1, w_c^2, \ldots , w_c^n)$ and the query sequence $Q=(w_q^1, w_q^2, \ldots , w_q^m)$, the input is formulated as a sequence $S_{q,c} =(\mathrm{[CLS]}, w_q^1, w_q^2, \ldots , w_q^m,$ $\mathrm{[SEP]}, w_c^1, w_c^2, \ldots , w_c^n,$ $\mathrm{[SEP]})$, where $\mathrm{[CLS]}$ indicates the start token of *Q* and $\mathrm{[SEP]}$ separates *Q* and *C*. Then, the word sequence input is tokenized to token sequence ${\mathbf{s}} ={[s_i]_{i=1}^k}$ concatenating with their position embedding and segment embedding. Denote the BERT encoder which consists of L stacks of transformers as $BERT(\cdot )$ as follows:

1 $$\begin{aligned} {\mathbf{s}} _i^l=Transformer({\mathbf{s}} _i^{l-1}),l \in [1,L] \end{aligned}$$

The hidden representation ${\mathbf{h}} ={[h_i]_{i=1}^k}$ for the token sequence obtained from BERT is ${\mathbf{h}} =BERT({\mathbf{s}} )$.

### Knowledge-enhanced attention layer

To obtain the knowledge-enhanced context representation, this layer is designed to integrate knowledge representation with the context representation of BERT. Here, we describe the details of this layer based on CID relation extraction. In the knowledge base, the same entity pair in different documents may contain different relation types. This layer shows how to integrate noisy knowledge representation into the context representation simply and effectively. It takes the BERT hidden representation ${\mathbf{h}}$ and the knowledge representation ${\mathbf{r}}$ as inputs, and outputs the knowledge-enhanced representation ${\mathbf{h}} ^{'}$.

**Fig. 2:**
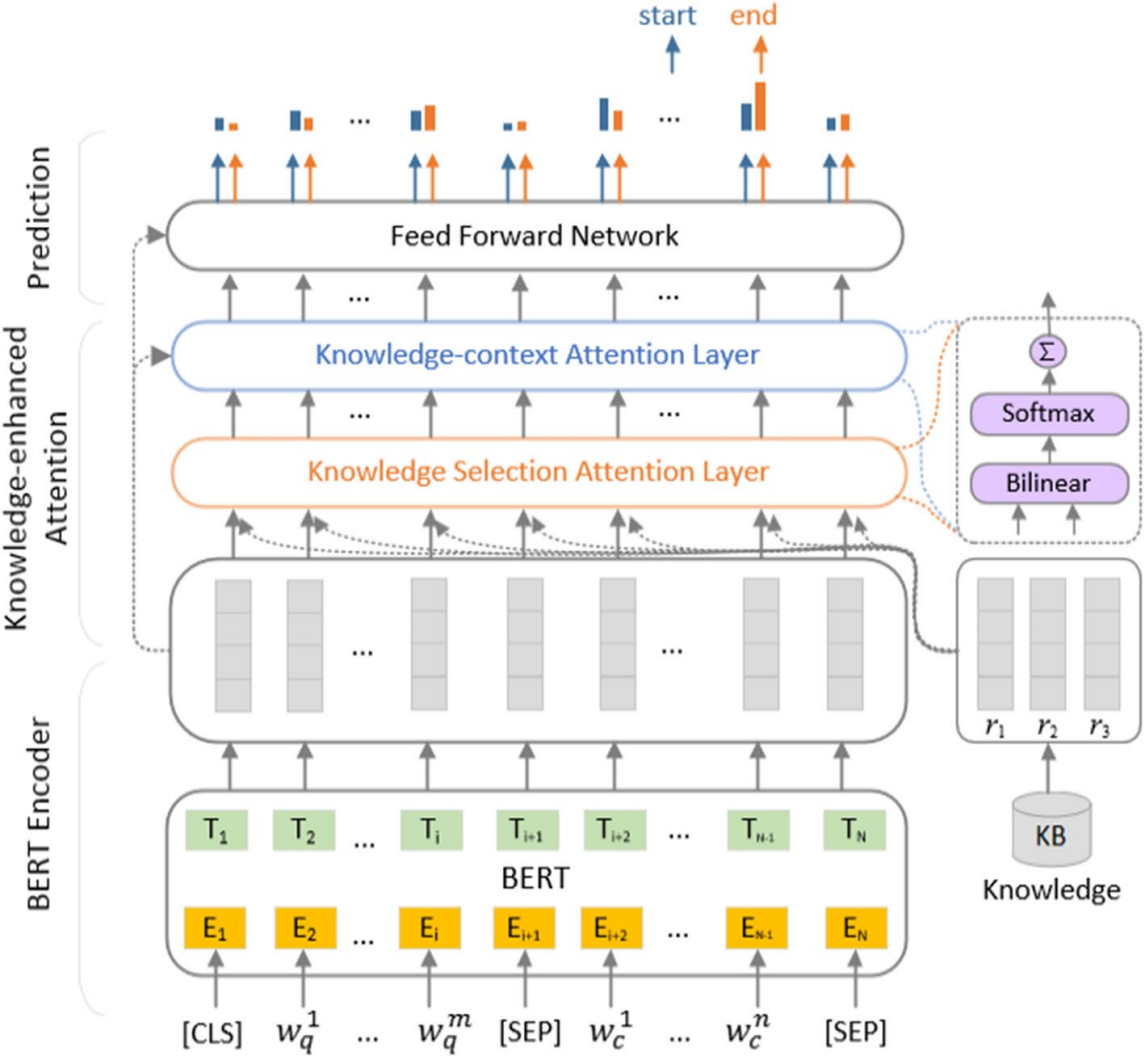
The overview of the knowledge-enhanced RC model

To integrate prior knowledge representation, we first extract chemical-disease triples from the Comparative Toxicogenomics Database(CTD) [18] and employ TransE [19] to learn knowledge representation. Following [1], we extract (*chemical, disease, relation*) triples from both the CDR corpus and the CTD knowledge base. In the CTD base, there are three types of relations, including ‘*marker/mechanism*’, ‘*therapeutic*’ and ‘*inferred-association*’, where only ‘*marker/mechanism*’ indicates the CID relation. For those pairs in CDR but not in CTD, we set their relations to a specific symbol *‘null’*. Thus, there are four types of relations among all the triples and we finally obtain 2577184 triples for knowledge representation learning. Then, all the generated triples are regarded as correct examples to learn lower-dimension chemical representation, disease representation and relation representation ${\mathbf{r}} _t$ by TransE, where ${\mathbf{r}} _t \in {\mathbb {R}}^{d_2}$, and $d_2$ denotes the representation dimension. Here, the chemical, disease and relation representations are initialized randomly with the normal distribution for training. It is worth noting that there may be more than one relation type between an entity pair (Fig. 2).

Then, the probable relation representation ${\mathbf{r}} =[{\mathbf{r}} _t]_{t=1}^3$ between candidate entities of each instance is introduced into the RC model to provide evidence. Here, we take two-step attention to combine the knowledge information and context information. First, we adopt an attention mechanism to select the most relevant KB relation representation for hidden representation of each token. A bilinear [20] operation is employed to calculate the attention weights between hidden representation ${\mathbf{h} }_i \in {\mathbb {R}}^{d_1}$ and relation representation ${\mathbf{r}} _t \in {\mathbb {R}}^{d_2}$, where ${\mathbf{W}} _{1} \in {\mathbb {R}}^{{d_1}\times {d_2}}$ and ${\mathbf{b}} _{1} \in {\mathbb {R}}^{d_2}$ are trainable parameters.

2 $$\begin{aligned} {\alpha _{it}}=\frac{exp({\mathbf{h}} _{i}{} {\mathbf{W} }_{1}{} {\mathbf{r}} _{t}+{\mathbf{b}} _{1})}{\sum _{t^{'}=1}^{3}exp({\mathbf{h}} _{i}{} {\mathbf{W}} _{1}{} {\mathbf{r}} _{t^{'}}+{\mathbf{b}} _{1})} \end{aligned}$$

Then, each relation representation ${\mathbf{r}} _t$ is aligned to each hidden state ${\mathbf{h}} _i$. Here, ${\mathbf{k}} _i$ is regarded as the weighted relation representation corresponding to each token.

3 $$\begin{aligned} {\mathbf{ k _{i}}}=\sum _{t}\alpha _{it}{} {\mathbf{r}} _{t} \end{aligned}$$

Second, we adopt a knowledge-context attention mechanism between the token’s knowledge representation ${\mathbf{k}} _{i}$ on each position of the token sequence and the hidden representation ${\mathbf{h}} _j$. A bilinear operation is employed between ${\mathbf{k} }_i$ and ${\mathbf{h}} _j$ to achieve weights on the hidden representation, while ${\mathbf{W}} _2 \in {\mathbb {R}}^{{d_2}\times {d_1}}$ and ${\mathbf{b}} _2 \in {\mathbb {R}}^{d_1}$ are parameters.

4 $$\begin{aligned} {\beta _{ij}}=\frac{exp({\mathbf{k}} _{i}{} {\mathbf{W}} _{2}{} {\mathbf{h}} _{j}+{\mathbf{b}} _{2})}{\sum _{j^{'}=1}^{k}exp({\mathbf{k}} _{i}{} {\mathbf{W}} _{2}{} {\mathbf{h}} _{j^{'}}+{\mathbf{b}} _{2})} \end{aligned}$$

Finally, the hidden representation ${\mathbf{h}}$ of tokens is aligned to the weighted knowledge representation ${\mathbf{k}}$ and weighted to each position *i*. Here, we denote the output after our two-step attention as ${\mathbf{h}} ^{'}=[{\mathbf{h}} _{i}^{'}]_{i=1}^{k}$.

5 $$\begin{aligned} \mathbf{ h _{i}^{'}}=\sum _{j}\beta _{ij}{} \mathbf{h} _{j} \end{aligned}$$

Here, ${\mathbf{h} }_{i}^{'}$ is the context representation enhanced with knowledge representation.

### Prediction layer

To obtain the final representation for prediction, the hidden representation $\mathbf{h} _i$ and the knowledge enhanced representation $\mathbf{h} _i^{'}$ are first combined with a linear operation to achieve the weighted representation $\mathbf{v} _i = \mathbf{W} _{h}{} \mathbf{h} _{i}+\mathbf{W} _{h^{'}}{} \mathbf{h} _{i}^{'}+\mathbf{b}$. Then, we concatenate the knowledge enhanced representation $\mathbf{h} _i^{'}$ with the weighted representation $\mathbf{v} _i$ to achieve the input $\mathbf{u} _i=[\mathbf{h} _i^{'}; \mathbf{v} _i]$ of the prediction layer. A feed-forward network FFN with RELU [21] activation is applied to the knowledge attention result, which works in some existing work. Finally, the output is applied to predict the start and end indexes of answers. For the situation where there is a null answer, its start and end indexes are both zero for the optimization of the objective function. Actually, the index of zero indicates the start token ‘[CLS]’. It is not the real text in the context and does not influence the optimization of the model for the indexes of non-null answers.

6 $$\begin{aligned} FFN(\mathbf{u} _{i}, \mathbf{W} _{3}, \mathbf{b} _{3}, \mathbf{W} _{4}, \mathbf{b} _{4}) = RELU(\mathbf{u} _{i}{} \mathbf{W} _{3}+\mathbf{b} _{3})\mathbf{W} _{4}+\mathbf{b} _{4} \end{aligned}$$

Here, $\mathbf{W} _{4}$, $\mathbf{b} _{4}$, $\mathbf{W} _3$ and $\mathbf{b} _3$ are trainable parameters. Along the sequence dimension, the *start* probability distribution and the *end* probability distribution for each token $\mathbf{s} _i$ are calculated as:

7 $$\begin{aligned} p_i^{s}= & {} \frac{exp(FFN(\mathbf{u} _{i}, \mathbf{W} ^s _3, \mathbf{b} ^s _3, \mathbf{W} ^s _4, \mathbf{b} ^s _4))}{\sum _{j}exp(FFN(\mathbf{u} _{j}, \mathbf{W} ^s _3, \mathbf{b} ^s _3, \mathbf{W} ^s _4, \mathbf{b} ^s _4))} \end{aligned}$$

8 $$\begin{aligned} p_i^{e}= & {} \frac{exp(FFN(\mathbf{u} _{i}, \mathbf{W} ^e _3, \mathbf{b} ^e _3, \mathbf{W} ^e _4, \mathbf{b} ^e _4))}{\sum _{j}exp(FFN(\mathbf{u} _{j}, \mathbf{W} ^e _3, \mathbf{b} ^e _3, \mathbf{W} ^e _4, \mathbf{b} ^e _4))} \end{aligned}$$

After answer prediction, a predicted disease text or a null answer can be achieved. If the predicted disease text matches the gold disease name, a CID relation will be detected between the disease and the chemical which is described in its corresponding question.

After relation extraction on intra-sentential and inter-sentential data, two sets of prediction results are merged. Since all the candidate instances with respect to mention pairs are extracted, we judge that an entity pair has a CID relation as long as at least one instance was detected in which the CID relation exists. Since several documents may have no candidate CID relations after data preprocessing, similar to many other systems, we take the following heuristic rules to find the likely CID pairs in them: All chemicals in the title are associated with all diseases in the abstract.

### Objective function

To predict the start and end index of answer spans, the optimization objective is to maximize the conditional probability $p(y_{s}, y_{e}|\mathbf{s} )$ of start index $y_{s}$ and end index $y_{e}$ on the given input sequence $\mathbf{s}$. The loss is defined as the average of log probabilities of the ground truth start and end position based on the predicted distributions. *N* is the number of examples. The answer span index by (*i*, *j*) with maximum $p_{i}^{s}p_{j}^{e}$ is chosen as the answer span.

9 $$\begin{aligned} Loss = -\frac{1}{N}\sum _{l=1}^{N}\frac{y_{s}log(p^{s})+y_{e}log(p^{e})}{2} \end{aligned}$$

## Experiments and results

### Dataset

We evaluated our model on two document-level biomedical relation extraction datasets, including the BioCreative V CDR dataset and the CHR dataset. Table 1 shows the overall statistics of the two datasets.

The BioCreative V ** CDR** dataset^1^ was derived from the Comparative Toxicogenomics Database (CTD) for CID relation extraction. The position and MeSH IDs of chemicals and diseases are annotated by experts. It contains 1500 titles and abstracts of PubMed articles, where the training, development and test sets each consist of 500 abstracts. Following [1], we combine the training set and development set together as a set due to the limited number of CDR examples, 80% of which is used as training and 20% of which is used as validation.

Experiments are also conducted on the ** CHR** dataset [2]. It was created by distant supervision and is a document-level dataset with relations between chemicals. It contains 12,094 titles and abstracts of PubMed articles, 7298, 3158 and 9578 each for training, development and test datasets.

**Table 1:** The overall statistics of the CDR and CHR datasets

Dataset	Splits	Documents	Chemical IDs	Disease IDs	pos	neg
CDR	Training	500	1479	1961	1038	4479
	Development	500	1519	1851	1012	4310
	Test	500	1455	2007	1066	4471
CHR	Training	7298	28158	–	19643	69843
	Development	1182	4575	–	3185	11466
	Test	3614	13800	–	9578	33339

The experimental results are evaluated by comparing the set of annotated chemical-disease relations in the document with the set of predicted chemical-disease through precision (P), recall (R) and F1-measure (F1).

### Data preprocessing

To transform data to RC format instances, data was preprocessed as follows.

** Instance Construction**  We extracted entity pair candidate instances from the original data, including intra-sentential and inter-sentential instances. For intra-sentential instances, all the entity pairs existing in the same sentence are extracted. For the inter-sentential instances, we follow some of the rules [1] to extract candidates: (1) In a document, all intra-sentential chemical-disease instances will not be considered as inter-sentential instances. (2) The sentence distance of all the inter-sentential instances will not be more than 3. Thus, the chemical-disease entity candidate pairs and their corresponding contexts are extracted. To be in line with the RC model, we will remove (mask) the other disease mentions except for the disease in the current pairs when multiple diseases occur in the context.

** Hypernym Filtering**  According to the annotation guideline of the CID task, it is to extract the most specific chemical-disease pair. Following [1], we remove the instances containing hyper entities that have more specific entities in the document according to the entity index in the Medical Subject Headings (MeSH) [22]. Some of positive chemical-disease entity pairs may be filtered by this strategy and are treated as false negative instances.

** Query Construction**  After extracting the chemical-disease candidate instances, we format a natural language query combining entity $e_{1}$ mention and relation *r* description, here *r* is the chemical-induced disease relation. Taking the candidate instance (*flunitrazepan*, *pain*) in $S_7$ in Fig. 1 as an example, we formulate a query *“what disease does flunitrazepan induce”* to ask the context expecting the answer is *pain*. Also, we adopt another strategy to format a pseudo query for comparison with the natural language query. We concatenate the entity $e_{1}$, the relation *r* description and the type of entity $e_{2}$ to construct the pseudo query.

On the ** CHR** dataset [2], all the chemical-chemical entity pairs and their full titles and abstracts are extracted for instance construction. After extracting the chemical candidate instances, queries were also constructed with the entity $e_{1}$ mention and relation *r* description. Here, *r* is the chemical reaction relation description.

### Implementation details

We trained our knowledge on TransE^2^ with 1000 epochs and the relation embedding size was set to 256. For the CDR dataset, we tuned the hyperparameters on the new development set to optimize our proposed model. We use the uncased BioBERT(base) as the pretrained language model. We set the batch size to 12, 32 respectively on the CDR dataset and the CHR dataset. The learning rates of the bert encoder and the downstream structure are set to 3e-5 and 1e-4 on the experiments without KBs, while their learning rates are set to 2e-5 and 3e-5 on the experiments with KBs.

### Compared models

To evaluate our approach, we compared the proposed model with the existing relevant models. As shown in Table 2, the comparison models for the CDR dataset were divided into two categories: methods with knowledge bases (KBs) and methods without knowledge bases. Each category includes traditional machine learning (ML) based methods and neural network (NN) based methods. Here, we mainly introduce the NN-based methods.

- *CNN+SDP*  [6] proposed using CNN to learns features on the shortest dependency path between a disease and a chemical for CID relation extraction.
- *BRAN (Transformer)*  [3] utilized an efficient transformer to encode abstracts and form pairwise predictions using a bi-affine operation to score all pairs of mentions and aggregating over mention pairs.
- *Bio-Seq (LSTM+CRF)*  [23] proposed a sequence labeling framework for biomedical relation extraction and extended it with multiple specified feature extractors, especially for inter-sentential level relation extraction.
- *LSTM+CNN*  [4] utilized LSTM and CNN to hierarchically extract from documents integrating relation knowledge of CTD.
- *RPCNN (PCNN)*  [5] proposed using PCNN and RNN to extract document-level representations integrating the knowledge features of four medical KBs for CID relation classification.
- *KCN (GCNN)*  [1] combined the shortest dependency path (SDP) sequence and knowledge representation for CID relation classification. It adopted the gated convolutional network (GCNN) with attention pooling combining entity and relation knowledge representations.
- *Ours*  Different from other models, we propose a new RC framework for biomedical relation extraction and utilize the pretrained LM combined with the knowledge of relation representation between the possible chemical and disease. It is worth noting that our model can automatically distinguish different types of relation knowledge from CTD.

### Performance comparison with previous methods

We compare our proposed model with previous work respectively on the CDR and CHR datasets.

For the ** CDR** dataset, we divide previous models into two categories: models without knowledge bases and models with knowledge bases. Here, the compared models are rich and diverse, such as heuristic rules, joint training with NER, relation classification, sequence labeling and so on.

Among the systems **without KBs**, much work is based on the neural networks (NNs). As shown in Table 2, the graph kernel-based SVM is competitive among the traditional ML methods, but most of NN-based methods outperform the traditional ML methods which indicates the NN’s more effective ability of context modeling. They almost use entity pair relation classification methods except that *Bio-Seq* [23] adopts sequence labeling to deal with the CID extraction task. Under the condition of no extra knowledge, our RC-based model outperforms the previous work and achieves an improvement of 0.19%. *Bio-Seq* [23] is a sequence labeling framework adopting LSTM and CRF. Compared with *Bio-Seq* [23], there is an improvement of 2.57%. *CNN+SDP* [6] is a relation classification method. It extracted the CID relation with CNN over the shortest dependency path (SDP) of context to deal with the long sequence and achieved an F1 score of 65.88%. Our method transforms the relation classification to reading comprehension, no matter on intra-sentential data or inter-sentential data. Different from *CNN+SDP* [6], our model does not need to transform the context sequence into SDP and just uses the pretrained LMs to extract context information directly from the context sequences. We conduct pointer prediction over the context sequences instead of classification and achieve the state-of-the-art performance of 66.07% on the CDR extraction data under the condition of no KBs.

**Table 2:** Doc-level performance comparison over our proposed model without and with knowledge on the CDR dataset

KBs	Model	P (%)	R (%)	F1 (%)
*Without KBs*				
Traditional ML	ME [8]	62.00	55.10	58.30
	Kernel-based SVM [24]	53.20	69.70	60.30
NN-based ML				
Relation classification	CNN+SDP [6]	58.02	76.20	65.88
	LSTM+CNN [25]	56.20	68.00	61.50
	BRAN(Transformer) [3]	55.60	70.80	62.10
	CNN+CNNchar [11]	57.00	68.60	62.30
	GCNN [2]	52.80	66.00	58.60
Sequence labeling	Bio-Seq(LSTM+CRF) [23]	60.00	67.50	63.50
Reading comprehension	RC (Ours)	65.83	66.32	** 66.07**
*With KBs*				
Traditional ML				
	SVM+Rules(+CTD)[26]	68.15	66.04	67.08
	SVM(+CTD+SIDER+MEDI+MeSH) [9]	65.80	68.57	67.16
	Kernel-based models(+CTD) [10]	60.84	76.36	67.72
	SVM(+Euretos KB) [27]	73.10	67.60	70.20
NN-based ML				
Relation classification	CAN(+CTD) [7]	60.51	80.48	69.08
	LSTM+CNN(+CTD) [4]	63.60	76.80	69.60
	RPCNN(+CTD+SIDER+MEDI+MeSH fea) [5]	65.24	77.21	70.77
	KCN(+CTD) [1]	69.65	72.98	** 71.28**
Reading comprehension	KRC(+DCh-Miner) (Ours)	65.33	67.17	66.23
	KRC(+CTD) (Ours)	71.93	70.45	** 71.18**

‘fea’ denotes features

In addition to the context information, the knowledge information also plays an important role in our RC-based model for CID relation extraction. Among the systems ** with KBs**, it mainly includes feature-based traditional ML methods and neural network (NN)-based methods. Most of the feature-based traditional ML methods adopt support vector machines (SVMs) and other kernel-based models. More details and differences of the compared NN-based methods can be seen in Section 4.4. Inspired by [1], we utilize the knowledge low-dimension representation to guide our RC-based model for chemical-induced disease extraction and then derive the CID relation. Different from [1], our method does not need to extract the SDP of the sequence and can integrate more than one type of relation from CTD into the RC model through the knowledge attention mechanism. On the NN-based models with knowledge representation, our KRC model achieves competitive performance. Compared with the *only KB* method and our RC model without KBs (*RC* in Table 2), our RC model with KBs (*KRC* in Table 2) performs better, which indicates that our KRC model can discern different knowledge and incorporate the knowledge with our attention layers effectively. To test our KRC model on other KBs, we also conduct experiments with the disease-drug association network (DCh-Miner)^3^ of the Stanford SNAP database. Compared with the performance without KBs, it is slightly better. Compared with the performance on CTD knowledge, it performs not so well. It may be caused by the difference between the two KBs. DCh-Miner only contains a relation that means the drug is associated with disease and we name it “association”, while the CTD knowledge contains three relations including “therapeutic”, “inferred-association”, “marker /mechanism” and only “marker /mechanism” indicates the relation of chemical-induced disease. It is worth noting that DCh-Miner is extracted from the CTD and the relation “association” covers three relations of CTD. The knowledge in DCh-Miner may mislead the model and decrease the performance.

**Table 3:** Doc-level performance comparison over our proposed model without knowledge on the CHR dataset

	Model	P (%)	R (%)	F1 (%)
*NN-based ML*				
Relation classification	CNN-RE [2]	81.2	87.3	84.1
	RNN-RE [2]	83.0	90.1	86.4
	GCNN [2]	84.7	90.5	87.5
Reading comprehension	RC (Ours)	93.5	93.0	**93.3**

For the ** CHR** dataset, there are only models without knowledge bases for comparison. Since it was created by distant supervision with the graph database Biochem4j and chemical relation labels in the dataset are the same as those in Biochem4j, we did not add this knowledge to our proposed model for prediction. As shown in Table 3, three previous NN-based methods are compared with our proposed model. *GCNN* [2] built a labeled edge graph convolutional neural network model on a document graph for document-level biomedical relation extraction. Different from *GCNN* [2], we transform the entity pair classification over a document to reading comprehension over a document. Compared with *GCNN* [2], we observe that our proposed RC model is 5.8 percentage points higher than the best F1 score and achieves the state-of-the-art performance.

## Discussion

### The effect of different pretrained models

As shown in Table 4, we compared models finetuned on extra open-domain reading comprehension data. Since our data is biomedical text, we choose the *biobert* [28] as the base model which is a language model named bert pretrained on a large scale of biomedical text. To investigate the effect of reading comprehension pretrained tasks, we further utilize the biobert model finetuned on the SQuAD [17] data by [29]. We named this model biobert+SQuAD in Table 4. Here, SQuAD is a large-scale open-domain reading comprehension dataset. Compared with both traditional ML methods and neural network based methods, our RC model based on biobert achieves the top 3 performance. It indicates that our proposed RC framework is effective for biomedical relation extraction. Compared with results only on the biobert model, adding open-domain reading comprehension data helps improve the performance of the CID relation extraction on the CDR data and there is an improvement of 1.87%. Benefitting from this RC framework, we can utilize large-scale data from an open domain reading comprehension task to help biomedical relation extraction especially when the biomedical relation extraction data is not enough.

**Table 4:** Results over LMs finetuned by open-domain reading comprehension dataset without KBs on the CDR dataset

Model	Intra sentential level			Inter sentential level			Document level		
	P (%)	R (%)	F1 (%)	P (%)	R (%)	F1 (%)	P (%)	R (%)	F1 (%)
biobert	62.27	** 57.13**	59.59	49.80	** 11.44**	18.61	59.85	** 69.23**	64.20
biobert+SQuAD	** 67.09**	54.50	** 60.14**	** 60.10**	11.16	** 18.83**	** 65.83**	66.32	** 66.07**

### The effect of query construction

As described in Section 4.2, two kinds of question construction methods are designed for our RC model. To select a better query construction strategy, we investigate the effect of these construction methods on performance. The experiments are conducted on our RC model based on the SQuAD finetuned biobert model under the condition of no knowledge. The performance on the pseudo query and the natural language query is shown in Table 5. The results show that using the natural language query achieves a higher document-level F1 of 66.07%. The reason may be that the natural query provides fine semantic hints for CID relation extraction while the pseudo query is just the simple concatenation of one entity mention, a relation type and another entity type which may confuse the model.

**Table 5:** Results over pseudo queries and natural queries without KBs on the CDR dataset. ‘Natural Query’ means natural language queries

Model	Intra sentential level			Inter sentential level			Document level		
	P (%)	R (%)	F1 (%)	P (%)	R (%)	F1 (%)	P (%)	R (%)	F1 (%)
Pseudo Query	66.75	52.91	59.03	57.66	** 12.01**	** 19.88**	64.90	65.57	65.24
Natural Query	** 67.09**	** 54.50**	** 60.14**	** 60.10**	11.16	18.83	** 65.83**	** 66.32**	** 66.07**

### The effect of knowledge representation

As shown in Table 6, we investigate the effect of knowledge combination, including *only KB*, *no KB(RC)*, and *adding KB*. In the part of adding KB, we compared two kinds of methods for knowledge-enhanced attention in our RC model. *Only KB* means we just directly match the relation of entity pairs in the CDR test set with the triples in CTD. From the result of only KB, we can see that the recall is high and the precision is not so well. It indicates that there is noise in triples extracted from CTD. Also, these triples can not fully cover CDR data. Thus, it is necessary to combine the CDR data and CTD knowledge in the RC model. Compared with the results of *only KB*, our proposed RC model performs better which indicates that the context information can be somehow captured by our RC model for CID relation extraction. As described in Section 3.2, there may be more than one KB relation representation between an entity pair. To further investigate the effect of our knowledge-enhanced RC model, we compared two operations of KB relation representation in the first step of our knowledge-enhanced attention layer. *RC+KB(atten2)* means we adopt the average operation for different KB relation representations. *RC+KB(atten1+atten2)* means we adopt the attention mechanism to automatically select the relevant KB relation representation in the model. The results on *RC+KB(atten2)* and *RC+KB(atten1+atten2)* show that the automatic attention selection works when more than one relation representation occurs and achieves a higher F1 of 71.18%. To further analyze the results of instances accompanied by different types of relations extracted from CTD, a detailed case study of no KBs and using KBs can be seen in the following section.

**Table 6:** Ablation study over our proposed KRC model on the CDR dataset

Model	Intra sentential level$^{\mathrm{1}}$			Inter sentential level$^{\mathrm{2}}$			Document level		
	P (%)	R (%)	F1 (%)	P (%)	R (%)	F1 (%)	P (%)	R (%)	F1 (%)
Only KB	58.61	** 62.29**	60.39	39.56	** 18.67**	** 25.37**	52.86	** 81.61**	64.16
RC	67.09	54.50	60.14	60.10	11.16	18.83	65.83	66.32	66.07
RC+KB(atten2)	67.94	56.85	61.90	62.00	11.63	19.59	66.88	69.14	67.99
RC+KB(atten1+atten2)	** 72.74**	58.07	** 64.58**	** 68.31**	11.73	20.02	** 71.93**	70.45	** 71.18**

$^{\mathrm{1,2}}$metrics on intra and inter sentential levels, we used the calculation methods in [1]. If using the calculation methods in [24], F1 measures of our model without KBs (*RC* in the table) are 73.82% and 57.07% respectively on intra and inter sentential levels

To investigate the robustness of our approach dealing with knowledge, we selected knowledge from the CTD knowledge base (KB) respectively for intra sentential and inter sentential data by four ratios, including 0.25, 0.5, 0.75, and 1.0 to conduct the knowledge-enhanced experiments on the CID task. The performance gradually increases with the increase of the knowledge proportion. When the selection ratio is 1.0, there is about 74% of data guided by CTD knowledge. When the selection ratio is 0.75, there is about 55% of data guided by CTD knowledge. As shown in Table 7, the performance of our KRC model surpasses that of our model without KBs and improves obviously when the selection ratio is more than 0.75, that is to say, the proportion of data guided by knowledge is more than 55%. Otherwise, it will decrease the performance.

**Table 7:** Doc-level performance on the CDR dataset with different scales of CTD knowledge

KB(CTD)$\_$Ratio	0.25	0.50	0.75	1.0
Document-level F1 (%)	63.68	64.81	67.09	71.18

### Case study of knowledge effect

This section analyzes relation complexity of integrating knowledge, good and bad cases with and without knowledge. According to the CTD guideline, there are three relation types in the knowledge base. Therefore, more than one candidate relation type can be extracted from CTD for an entity pair in some cases and they may be consistent or inconsistent with the true relation type of the entity pair.

**Fig. 3:**
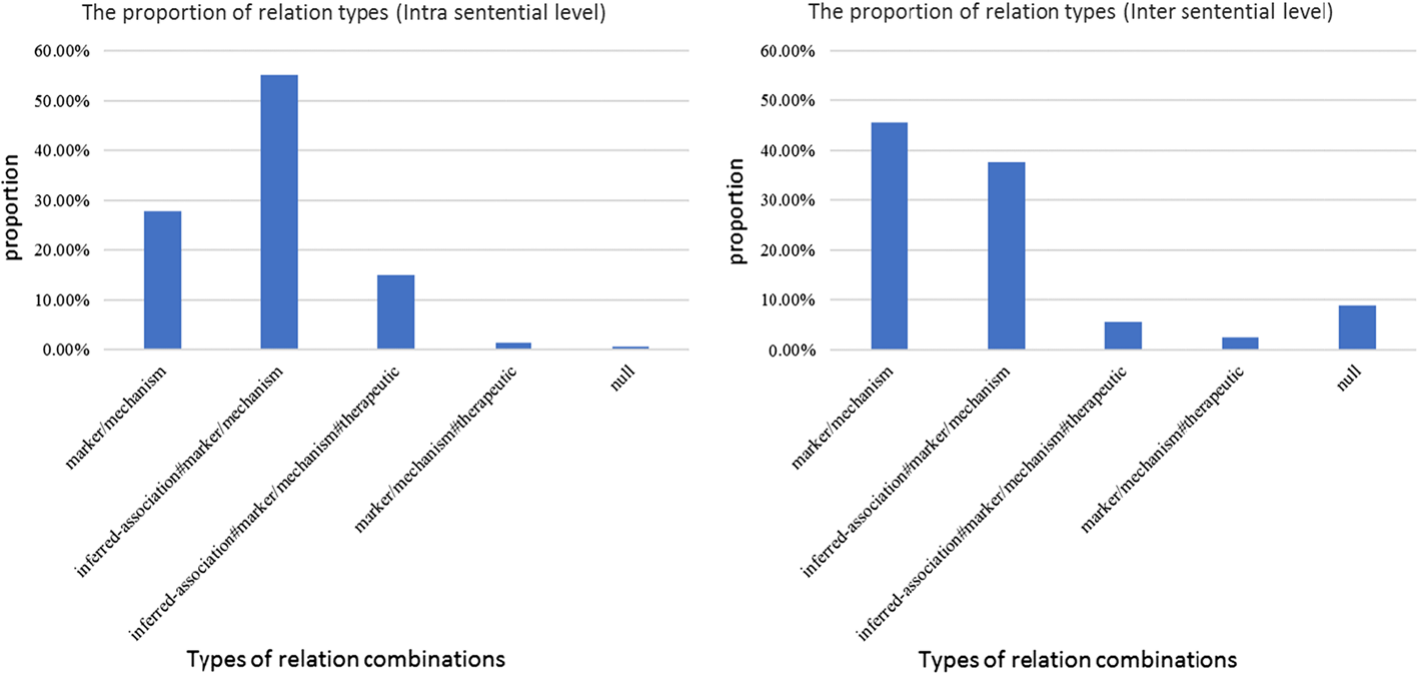
The proportion of relation combination types extracted from CTD in correctly predicted cases on the intra sentential and inter sentential level

To discuss the relation complexity when integrating knowledge, we counted the proportion of different relation combinations extracted from CTD for corrected extracted CID pairs as shown in Fig. 3. Here, *‘inferred-association’* means chemicals are inferred associated with diseases via CTD-curated chemical-gene interactions. *‘marker/mechanism’* indicates that a chemical may cause a disease. *‘therapeutic’* means a chemical that has a known or potential therapeutic role in a disease. * ‘#’* is a separator. The extracted relations to guide prediction for each CID pair are complex. Some of them are composed of more than one relation. *‘inferred-association#marker/mechanism’* contains two potentially related relation types. *‘marker/mechanism#therapeutic’* contains two conflict relation types. *‘inferred-association#marker/mechanism#therapeutic’* contains both related and conflict relation types. Except for the related relation types, the statistics in Fig. 3 show that our method can also deal with the cases with some noisy relation knowledge and extract the correct relations for entity pairs.

**Table 8:**
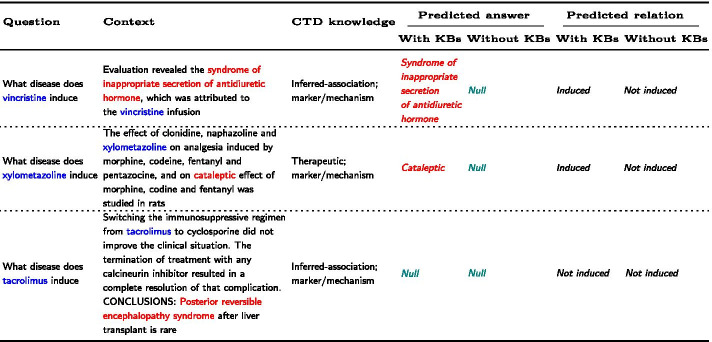
Good cases and bad cases on our RC model with and without KBs

Chemical and disease mentions are marked in blue and red respectively. Incorrect predicted answers are marked in teal. The gold answers of instances in line 1, line 2 and line 3 are ‘*syndrome of inappropriate secretion of antidiuretic hormone*’, ‘*cataleptic*’ and ‘*Posterior reversible encephalopathy syndrome*’ respectively. The gold relations of instances in line 1, line 2 and line 3 are ‘*induced*’

Additionally, we analyze some cases with different relation types extracted from CTD. As shown in Table 8, in the first example, the two relation types in CTD are potentially related. In this case, the CTD knowledge is consistent with the true relation type of the candidate entity pair. According to the CTD knowledge, *‘inferred-association’* and *‘marker/mechanism’*, our KRC model extracted the answer *‘syndrome of inappropriate secretion of antidiuretic hormone’* for the vincristine-induced disease correctly and predicted that vincristine induces the syndrome of inappropriate secretion of antidiuretic hormone, while our RC model without KBs extracted no answer and could not detect the CID relation in this case.

In the second example, we explore the case with two conflict relation types in CTD. In this case, *‘therapeutic’* is inconsistent with the true relation type of the candidate entity pair. Integrating two different relation types, our KRC model learned from the context and successfully picked the most relevant relation, predicting that the answer to the xylometazoline-induced disease was *‘cataleptic’*. While integrating no KBs, our RC model predicted that there was no answer in the context for the naphazoline-induced disease.

There are still implicit instances that are difficult for our model to extract disease spans, although the relation types from CTD knowledge indicate that the chemical may cause the disease. Take the context *“Switching the immunosuppressive regimen from tacrolimus to cyclosporine did not improve the clinical situation. The termination of treatment with any calcineurin inhibitor resulted in a complete resolution of that complication. CONCLUSIONS: Posterior reversible encephalopathy syndrome after liver transplant is rare.”* of the third case as an example. When asked by a query *“what disease does tacrolimus induce?”*, an answer *“Posterior reversible encephalopathy syndrome”* is expected to be extracted to indicate the CID relation between the chemical and the disease. However, no answer (*“null”*) was predicted. Here, the context did not obviously reveal the CID relation between *“tacrolimus”* and *“Posterior reversible encephalopathy syndrome”*. It just implies that the termination of the calcineurin inhibitor *“tacrolimus”* results in a complete resolution of that complication *“Posterior reversible encephalopathy syndrome”*, which indicates the CID relation between the two entities.

### Error analysis

**Fig. 4:**
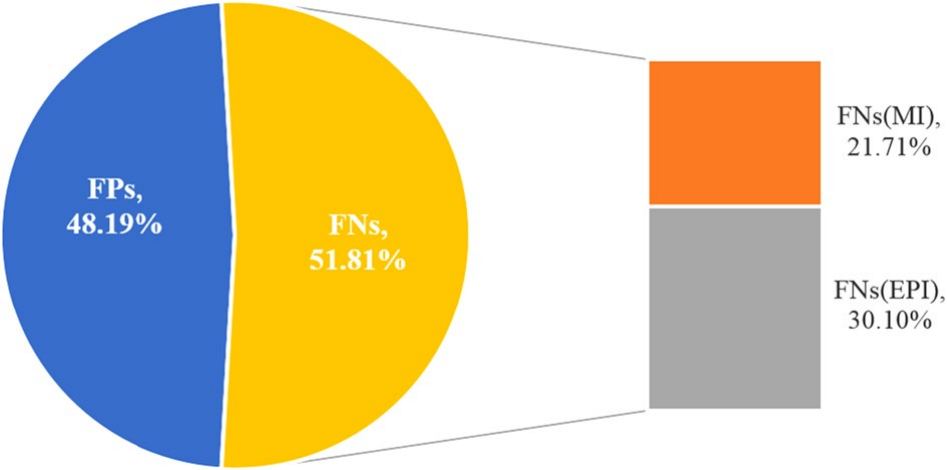
The error distribution. *FNs* denotes the false negative examples. *FPs* denotes the false positive examples. *FNs(MI)* denotes the missing instances for predicting in *FNs*. *FNs(EPI)* denotes the error predicted instances in *FNs*

To detect the main error sources, we performed error analysis on the final results of our proposed model on the CDR data as shown in Fig. 4. Among these errors, 293 negative chemical-disease entity pairs were wrongly classified as positive, accounting for 48.19%. In these instances, disease mentions were extracted by our KRC model while actually no answer should be predicted. Some inconsistent annotations may lead to some incorrectly annotated instances. Some curated CTD knowledge may mislead the predictions. Some instances containing complex context may make it difficult for our KRC model to extract the correct chemical-induced disease. Taking the sentence *“Ethambutol-induced toxic optic neuropathy was suspected and tablet ethambutol was withdrawn.”* as an example, *‘induced’* appears near *‘Ethambutol’* and *‘toxic optic neuropathy’* which may mislead the model to extract *‘optic neuropathy’* as the ethambutol-induced disease instead of the null answer.

315 positive chemical-disease entity pairs were wrongly classified as negative, accounting for 51.81%. Some positive instances were removed by the instance construction rules and hypernym filtering and they did not appear to be predicted by the model, which resulted in 132 errors accounting for 21.71%. In some other positive instances, no answer was extracted while actually disease mentions should be extracted. Taking the sentence *“METHODS: Newborn piglets received levobupivacaine until cardiovascular collapse occurred.”* as an example, there is no obvious trigger and expression about the relation *‘induce’* which may make it difficult to extract the levobupivacaine-induced disease *‘cardiovascular collapse’*. Besides, a few cases with the relation type *‘null’* of CTD knowledge led to incorrect prediction.

## Conclusions

In this paper, we propose a novel knowledge-enhanced reading comprehension framework for biomedical relation extraction, incorporated with an effective knowledge-enhanced attention mechanism to combine noisy knowledge. In the RC framework, it shows open-domain reading comprehension data and knowledge representation can significantly improve the performance of biomedical relation extraction. The experiments on the CDR data and the CHR dataset show that our proposed model achieved competitive F1 values of 71.18% and 93.3%, respectively, compared with other methods. In the future, we would like to design a more sophisticated reading comprehension model for biomedical relation extraction and apply it to other more complex biomedical tasks.

## Data Availability

The BioCreative V chemical disease relation (CDR) dataset can be downloaded at: https://biocreative.bioinformatics.udel.edu/tasks/biocreative-v/track-3-cdr/. The chemical reaction (CHR) dataset can be downloaded at: http://nactem.ac.uk/CHR/

## Abbreviations

RC: Reading comprehension
CID: Chemical-induced disease
CDR: Chemical disease relation
CHR: Chemical reaction
KBs: Knowledge bases
ML: Machine learning
NN: Neural network
ME: Maximum entropy
SVM: Support vector machine
SDP: Shortest dependency path
CNN: Convolutional neural network
LM: Language model
RE: Relation extraction
CTD: Comparative toxicogenomics database
MeSH: Medical subject headings

## Footnotes

1 https://biocreative.bioinformatics.udel.edu/tasks/biocreative-v/track-3-cdr/.

2 https://github.com/thunlp/KB2E/.

3 http://snap.stanford.edu/biodata/datasets/10004/10004-DCh-Miner.html.
